# Health professionals and students’ experiences of reflective writing in learning: A qualitative meta-synthesis

**DOI:** 10.1186/s12909-021-02831-4

**Published:** 2021-07-22

**Authors:** Giovanna Artioli, Laura Deiana, Francesco De Vincenzo, Margherita Raucci, Giovanna Amaducci, Maria Chiara Bassi, Silvia Di Leo, Mark Hayter, Luca Ghirotto

**Affiliations:** 1 Azienda USL-IRCCS di Reggio Emilia, Viale Umberto I, 50, 42123 Reggio Emilia, Italy; 2 Medical and Surgical Department, University of Parma, Parma, Italy; 3 European University of Rome, Rome, Italy; 4 Faculty of Health Sciences, University of Hull, Hull, UK

**Keywords:** Health care education, reflective writing, Health professionals, Health students, qualitative meta-synthesis

## Abstract

**Background:**

Reflective writing provides an opportunity for health professionals and students to learn from their mistakes, successes, anxieties, and worries that otherwise would remain disjointed and worthless. This systematic review addresses the following question: “What are the experiences of health professionals and students in applying reflective writing during their education and training?”

**Methods:**

We performed a systematic review and meta-synthesis of qualitative studies. Our search comprised six electronic databases: MedLine, Embase, Cinahl, PsycINFO, Eric, and Scopus. Our initial search produced 1237 titles, excluding duplicates that we removed. After title and abstract screening, 17 articles met the inclusion criteria. We identified descriptive themes and the conceptual elements explaining the health professionals’ and students’ experience using reflective writing during their academic and in-service training by performing a meta-synthesis.

**Results:**

We identified four main categories (and related sub-categories) through the meta-synthesis: reflection and reflexivity, accomplishing learning potential, building a philosophical and empathic approach, and identifying reflective writing feasibility. We placed the main categories into an interpretative model which explains the users’ experiences of reflective writing during their education and training. Reflective writing triggered reflection and reflexivity that allows, on the one hand, skills development, professional growth, and the ability to act on change; on the other hand, the acquisition of empathic attitudes and sensitivity towards one’s own and others’ emotions. Perceived barriers and impeding factors and facilitating ones, like timing and strategies for using reflective writing, were also identified.

**Conclusions:**

The use of this learning methodology is crucial today because of the recognition of the increasing complexity of healthcare contexts requiring professionals to learn advanced skills beyond their clinical ones. Implementing reflective writing-based courses and training in university curricula and clinical contexts can benefit human and professional development.

**Supplementary Information:**

The online version contains supplementary material available at 10.1186/s12909-021-02831-4.

## Background

Education of healthcare professionals supportstheir transformation into becoming competent professionals [1] and improves their reasoning skills in clinical situations. In this context, reflective writing (RW) is encouraged by both universities, and healthcare training providersencourage reflective writing (RW) since its utility in helping health students and professionals nurture reflection [2], which is considered a core element of professionalism. Furthermore, the ability to reflect on one’s performance is now seen to be a crucial skill for personal and professional development [3]. Writing about experiences to develop learning and growth through reflection is called ‘reflective writing’ (RW). RW involves the process of reconsidering an experience, which is then analyzed in its various components [4, 5]. The act of transforming thoughts into words may create new ideas: the recollection of the experience to allow a deeper understanding of it, modifying its original perception, and creating new insights [6]. RWis the focused and recurrent inspection of thoughts, feelings, and events emerging from practice as applied to healthcare practice [7].

Reflection may be intended as a form of mental processing or thinking used by learners to fulfill a purpose or achieve some anticipated outcome [2]. This definition recalls Boud and colleagues’ view of reflection as a purposive activity directed towards goals [8]. For those authors, reflection involves a three-stage process, including recollection of the experience, attending to own feelings, and re-evaluating the experience. This process can be facilitated by reflective practices, among which RW is one of the main tools [9].

Between reflection-on-action (leading to adjustments to future learning and actions) and reflection-in-action (where adjustments are made at the moment) [10], RW can be situated in the former. It involves theprofessional’s reflections and analysis of experiences in clinical practice [11, 12]. Mainly,RWinvolves the recurrent introspection ofone’s thoughts, feelings, and events within a particular context [13]. Several studies highlight how RWinfluencespromoting critical thinking [14], self-consciousness [15], and favors the development of personal skills [16], communication and empathy skills [4, 17], and self-knowledge [3]. Thanks to the writing process, individuals may analyze all the components of their experience and learn something new, giving new meanings [5]. Indeed, putting down thoughts into words enables the individual to reprocess the experience, build and empower new insights, new learnings, and new ways to conceive reality [6, 18–20].

Furthermore, RW provides an opportunity to give concrete meaning to one’s inner processes, mistakes, successes, anxieties, and worries that otherwise would remain disjointed and worthless [21, 22]. The reflective approach of RW allows oneself to enter the story, becoming aware of our professional path, with both an educational and therapeutic effect [23].

Reflection as practically sustained by RW commonly overlaps with the process of reflexivity. As noted elsewhere [24], reflection and reflexivity originate from different philosophical traditionsbut have shared similarities and meanings. In the context of this article, we adopt two different working definitions of reflection and reflexivity. Firstly, we draw from the work of Alexander [25]: who explains reflection as the deliberation, pondering, or rumination over ideas, circumstances, or experiences yet to be enacted, as well as those presently unfolding or already passed [25]. Reflexivity at a meta-cognitive level relates to finding strategies to challenge and questionpersonal attitudes, thought processes, values, assumptions, prejudices, and habitual actions to understand the relationships’ underpinning structure with experiences and events [26]. In other words, reflexivity can be defined as “the self-conscious co-ordination of the observed with existing cognitive structures of meaning” [27].

Given those definitions,a philosophical framework for helping health trainees and professionals conduct an exercise that can be helpful to them, their practice, and – ultimately – their patients can be identified. There is a growing body of qualitative literature on this topic – which is valuable – but the nature of qualitative research is that it creates transferrable and more generalizableknowledge cumulatively. As such, bodies of qualitative knowledge must besummarized and amalgamated to provide a sound understanding of the issues – to inform practice and generate the future qualitative research agenda. To date, this has not been done for the qualitative work on reflective writing: a gap in the knowledge base our synthesis study intends to address by highlighting what connects students and professionals while using RW.

This systematic review addresses the following question: “What are the experiences of health professionals and students in applyingRWduring their education and training?”

## Methods

This systematic review and meta-synthesis followed the 4-step procedure outlined by Sandelowski and Barroso [28, 29], foreseeing a comprehensive search, appraising reports of qualitative studies, classification of studies, synthesis of the findings. Systematic review and meta-synthesis referto the process of scientific inquiry aimed at systematically reviewing and formally integrating the findings in reports of completed qualitative studies [29].

The article selection processwas summarized as a PRISMA flowchart [30]; the search strategy was based on PICo (Population, phenomenon of Interest, and Context),and the study results are reported in agreement with Enhancing Transparency in Reporting the Synthesis of Qualitative Research (ENTREQ) guidelines [31].

### Selection criteria

Inclusion criteria for the meta-synthesis were:

- Primary qualitative studies published in peer-reviewed English journals.
- With health professionals or health studentsas participants.
- UsingRW in learning contexts (both pre-and in-service training).
- Mixed methods where the qualitative part can be separated.
- Articles should report the voice of participants (direct quotations).

Given the meta-synthesis indications, we excluded quantitative studies, non-primary research articles, meta-synthesis of qualitative studies, literature and systematic reviews, abstracts, unpublished reports, grey literature. In addition, we also excluded studies where participants were using RW in association with other learning tools and where the personal experience was not about using RW exclusively.

### Data sources and searches

An experienced information specialist (MCB) performed the literature search on Medline, Embase, Cinahl, PsycInfo, Eric, and Scopus for research articles published from Jan 1st, 2008 to September 30th, 2019,to make sure we incorporated studies reflecting contemporary professional health care experience. Additional searchinginvolved reviewing the references or, and citations to, our included studies.

We filled an Excel file with all the titles and authors’ names. A filter for qualitative and mixed methods study was applied. Table 1 shows the general search strategy for all the databases based on PICo.

**Table 1:** Search strategy for databases based on PICo

	MedLine	EMBASE	CINAHL	PsycINFO	Scopus
**P**	Health Personnel”[Mesh] OR psychologist*	psychologist* OR health professional*	(MH“Health Personnel”) OR psychologist*	exp Health Personnel/ (psychologist* or health professional*)	(psychologist* OR health AND professional*)
**I**	Autobiography as Topic”[Mesh] OR “Writing”[Mesh] OR writing* OR autobiographical*	autobiograph*: ab,ti OR writing: ab,ti	(MH“Writing”) OR (MH“Autobiographies”) OR (autobiographic* OR writing)	exp Autobiography/exp. Creative Writing/	(writing OR autobiography OR autobiographic*)
**Co**	Learning”[Mesh] OR “Education”[Mesh] OR “Thinking”[Mesh]	‘education’/exp./mj OR ‘thinking’/exp./mj	(MH“Thinking”) (MH“Learning”) OR (MH“Education”)	(MH“Thinking”) (MH“Learning”) OR (MH“Education”)	(training OR awareness OR critical AND thinking OR learning OR education)

* truncation

Four reviewers (GAr, MR, GAm, LD) independently screened titles and abstracts of all studies, then checked full-text articles based on the selection criteria. We also searched the reference lists of the full-text articles selected for additional potentially relevant studies. Any conflict was solved through discussion with three external reviewers (LG, MCB,SDL, and MH).

### Quality appraisal

We used the Critical Appraisal Skills Programme (CASP): it provides ten simple guiding questions and examples to examine study validity, adequacy, and potential applicability of the results of qualitative studies. Guided by the work of Long and colleagues [32] and previously used in other meta-synthesis [33], we created 30 items from the 10 CASP questions on quality to ensure we could provide a detailed appraisal of the studies. FDV and LD independently assessed the quality of included studies with any conflicts solved by consulting a third reviewer (MCB and LG). Researchers scored primary studies weighingthe proposed items and ranking the quality of each included study [34] on high (*n* > 20 items positively assessed), moderate (10 < *n* < 20), or low quality (*n* < 10).

### Analysis and synthesis

MCB created a data extraction table, GAr, GAm, and MRdescribed the included articles (Table 2). Quotations were extracted manually from the “results/findings” sections of the included studies by GAr, MCB, LDand inserted into adatabase. GAr, GAm, MR, and FDVperformed a thematic analysis of those sections, along with participants’ quotations. Then, they inductively derived sub-themes from the data, performing a first interpretative analysis of participants’ narratives (i.e., highlighting meanings participants interpreted about their experience). The sub-themes were compared and transferred across studies by adding the data into existing sub-themes or creating new sub-themes. Similar sub-themes were then grouped into themes, using taxonomic analysisto conceptually identify the sub-categories and the categories emerging from the participants’ narratives. This procedure allowed us to translate the themes identified from the original studies [28] into interpretative categories that could amalgamate and refine the experiences of health professionalsor health students on the use of RW [29]. The final categories are based on the consent of all the authors.

**Table 2:** Summary of articles included in meta-synthesis (divided per groups: students and professionals)

Source and country	Purpose	Sampling	Previous training on RW	Type of professionals	Method	Data collection	CASP
**STUDENTS**							
Tsang et al. (2010) [35] “Oral health students’ perceptions of clinical reflective learning-relevance to their development as evolving professionals” (Australia)	Student perceptions of clinical reflective learning and its relevance to their clinical and professional development.	17 students	Yes	Oral health professionals	Quantitative and qualitative analyses	Thematic analysis	14/30 Low to Moderate
Wald et al. (2010) [36] “The loss of my elderly patient: interactive reflective writing to support medical students’ rites of passage” (United States of America)	Implement a narrative medicine curriculum innovation of students’ reflective writing.	25 students	Yes	Doctors	Qualitative study	Brown Educational Guide to the Analysis of Narrative (BEGAN)	18/30 Moderate
Garrison et al. (2011) [37] “Qualitative analysis of medical student impressions of a narrative exercise in the third-year psychiatry clerkship” (United States of America)	Examine students’ written reactions to the narrative exercise, which drawing from narrative medicine and narrative therapy.	46 students	Yes	Doctors	Qualitative method	Thematic analysis	20/30 Moderate
Kuo et al. (2011) [38] “Using clinical caring journaling: nursing student and instructor experiences” (Taiwan)	Explore the experiences and perceptions of student nurses using clinical care journaling.	880 students + 90 clinical instructors	Yes	Nurses	Descriptive qualitative research	Constant comparative method	18/30 Moderate
Bagnato et al. (2013) [39] “The reflective journal: a tool for enhancing experience-based learning in nursing students in clinical practice” (Italy)	Understand the level of students’ reflections; The students’ experience.	33 students	Not described	Nurses	Qualitative data analysis	Mezirow’s qualitative method	13/30 Low to Moderate
Constantinou et al. (2013) [40] “Physiotherapy students find guided journals useful to develop reflective thinking and practice during their first clinical placement: a qualitative study” (Australia)	Do physiotherapy students perceive that guided journals facilitate reflective thinking and practice?	90 students	Yes	Physiotherapists	Mixed methods study	Leximancer© V3.5 Software	15/30 Low to Moderate
Jonas-Dwyer et al. (2013) [41] “First reflections: third-year dentistry students’ introduction to reflective practice” (Australia)	Introduce reflective practice to students; evaluate students’ self-perceived reflective skills before and after their reflective activities.	46 students	Yes	Dentists	Qualitative study	Wong et al.’s Schema	21/30 Moderate to High
Bowman et al. (2014) [42] “Academic reflective writing: a study to examine its usefulness” (United Kingdom)	To explore students’ experiences of doing assessed academic reflective writing.	8 students	Not described	Nurses and midwives	Qualitative research methodology	Kitzinger and Barbour’s method	19/30 Moderate
Padykula (2016) “RN-BS students’ reports of their self-care and health-promotion practices in a holistic nursing course” (United States of America)	Explore the utility of reflective journal writing for enhancing RN-BS students’ self-care and health-promotion practices.	15 students	Not described	Nurses	Qualitative single case study	Creswell’s method	26/30 High
Binyamin (2018) [43] “Growing from dilemmas: developing a professional identity through collaborative reflections on relational dilemmas” (Israel)	Illustrate how the pedagogical method of collaborative reflection can develop occupational therapists’ professional identity.	196 students	Yes	Occupational therapists	Qualitative research	Thematic analysis	16/30 Moderate
Hwang (2018) [44] “Facilitating student learning with critical reflective journaling in psychiatric mental health nursing clinical education: a qualitative study” (Korea)	Explore types of events or issues that senior nursing students chose to reflect upon in their critical reflective journals during their 5-week psychiatric mental health nursing clinical practicum; assess students’ evaluations of critical reflective journaling.	59 students	Yes	Nurses	Qualitative study	Qualitative content analysis	16/30 Moderate
Persson et al. (2018) [45] “Midwifery students’ experiences of learning through the use of written reflections – an interview study” (Sweden)	Examine how midwifery students experienced the writing of daily reflections on their practice.	19 students	Yes	Midwives	Interview study using an inductive method with descriptive design	Qualitative thematic content analysis.	23/30 Moderate to High
**PROFESSIONALS**							
Levine et al. (2008) [46] “The impact of prompted narrative writing during internship on reflective practice: a qualitative study” (United States of America)	Understand if prompted narrative writing led to increasing reflection by the study participants and what impact this had on participants’ attitudes and behaviors.	32 professionals	Not described	Internal medicine residents	Prospective qualitative study	Qualitative analysis	21/30 Moderate to High
Cashell (2010) [47] “Radiation therapists’ perspective of the role of reflection in clinical practice” (Canada)	To explore radiation therapist’s understanding of the concept of reflection and how it was incorporated into their daily practice.	123 professionals	Yes	Radiation therapists	Mixed methods study	Thematic analysis	21/30 Moderate to High
Vachon et al. (2010) [48] “Using reflective learning to improve the impact of continuing education in the context of work rehabilitation” (Canada)	Describe how occupational therapists used reflective learning to integrate research evidence into their clinical decision-making process and identify the factors that influenced the reflective learning process.	8 professionals	Yes	Occupational therapists	Collaborative research	The data analysis process was based on the methods proposed in Grounded Theory	25/30 High
Karkabi et al. (2014) [49] “The use of abstract paintings and narratives to foster reflective capacity in medical educators: a multinational faculty development workshop” (Israel)	Foster reflective capacity using art and narrative.	23 professionals	Yes	Family medicine physicians	Qualitative assessment	Thematic analysis	16/30 Moderate
Caverly et al. (2018) [50] “Qualitative evaluation of a narrative reflection program to help medical trainees recognize and avoid overuse” (United States of America)	To describe a writing program and to explore how participating influenced the thinking, attitudes, and behaviors.	20 professionals	Yes	Internal medicine residents	Qualitative research methodology	Thematic analysis	20/30 Moderate

## Results

### Literature search and studies’ characteristics

**Fig. 1:**
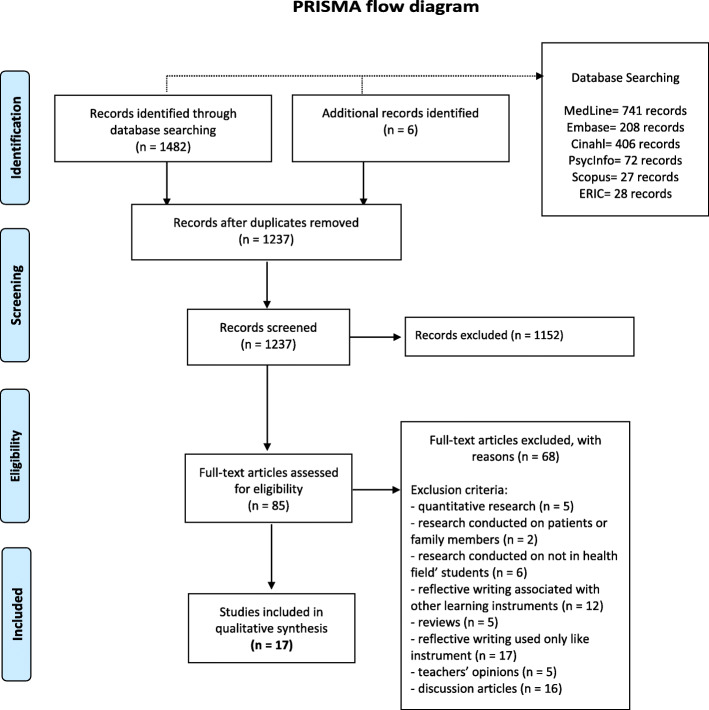
PRISMA flow diagram

A total of 1488 articles were retrieved. Duplicates (*n* = 251) were removed. Then, articles (*n* = 1237) were identified and reviewed by title and abstract. We excluded *n* = 1152 articles because they did not match the specified inclusion criteria, based on the title and abstract. Consequently, we assessed 85 full-text articles. Sixty-eight records did not meet the inclusion criteria. At the end of the selection process, 17 reportsof qualitative research were selected. Figure 1 illustrates the search process.

Table 2 shows the characteristics of the included studies. Eleven studies involved healthcare students (58%, including nurses, midwives, physiotherapists, doctors, dentists, and oral health students), and six (32%, including doctors, occupational and radiation therapists) were referred to health professionals. In thirteen studies, participants were trained on RW before using it: this information could not be retrieved from the remaining articles.

Five articles reported studies conducted in the US, three in Australia, two in Canada, and two in Israel. The other studies were carried out in Italy, UK, Korea, Taiwan, and Sweden.

### Critical appraisal results

We critically evaluatedall 17 studies to highlight the methodological strengthsand weaknesses of the selected studies. No article was removed on a quality assessment basis. Results of the quality appraisal are reported in Table 2.

### Meta-synthesis findings

Through the meta-synthesis, we identified four main categories (and related sub-categories): (i) reflection and reflexivity; (ii) accomplishing learning potential; (iii) building a philosophical and empathic approach; (iv) identifying reflective writing feasibility (for the complete dataset, please refer to supplemental material, where we have listed a selection of meaningful quotations of categories and sub-categories).

**Fig. 2:**
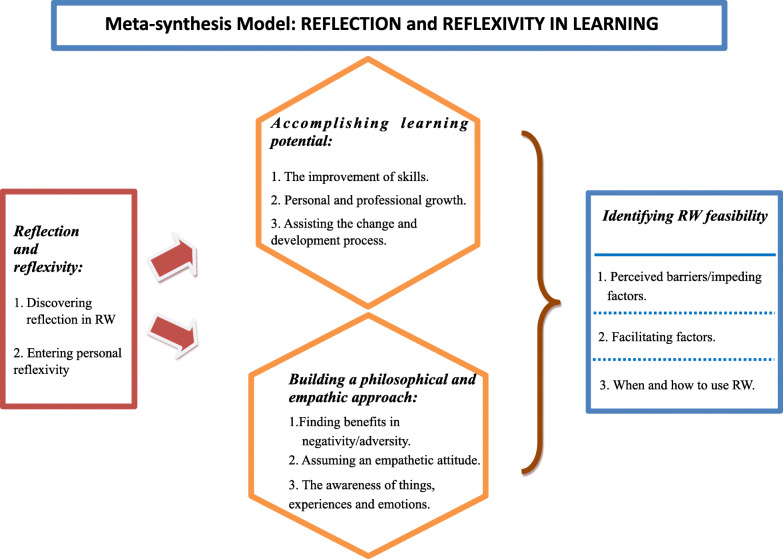
Meta-synthesis model: RW as experienced by health professionals and students

Given such categories, we developed an interpretative meta-synthesis model (Fig. 2) to illustrate the commonalities of the experience of using RW according to both students and professionals: RWas a vehicle for discovering reflection and allowing users to enter personal reflexivity to fulfillone’s learning potential, alongside the building of a philosophical and empathic approach. In their experience, reflection and reflexivity generate different skills and competencies: reflection matures skills such as professional skills and the ability to activate change and innovation. Reflexivity allows students and professionals to reach higher levels of competencyconcerning inner development and empathy reaching. Finally, from our analysis, participants, while recognizing the value of RW, also defined factors that could encourage or limit its use. Differences among participants’ groups are also outlined.

#### Reflection and reflexivity

Within this category, we collected the users’ narratives about the experience of applying RW and its disclosing capacity. By using RW, participants confronted themselves with both reflection and reflexivity. This category includes two sub-categories we named: discovering reflection and entering personal reflexivity.

##### Discovering reflection

The sub-category shows that experiencingRW deepened their reflection on experiences, practice, and profession. Thanks to RW, professionals, and students could explore previously unexplored topics and learn more about themselves.

“*Writing initiated me to think about my experiences …* ” (professional) [46].

“*I think it’s good for physicians to reflect on what we’re doing*” (professional) [50]

The analysis showed that RW was considered reflective when it provided an opportunity for those who applied it to stop, reflect and conduct an inner discourse on topics never considered before [44, 46, 50]. Some students affirmed:

“*Helped (me) reflect on positive aspects*” (student) [40].

“*I don’t usually think too much about what happens to me, but through critical reflective journaling, I was able to think carefully about things happening around me. This activity helped me to look into my mind*” (student) [44]

This sub-category explains transversal meanings coming from uniformly professionals and students.

##### Entering personal reflexivity

This sub-category includes data about RW enabling users’reflexivity. In this context, RW was considered training for reflexivity as it enabled participants to question themselves more often [48], reflect on their experiences [35], attitudes, actions [38, 45], and also reconsider their actions and identify their strengths and weaknesses [40, 44].

“*The questions in this study do make me stop and think about things – how I feel about what I’m doing in residency*”(professional) [46].

“*Helped me ID (identify) my strengths and weaknesses*” (student) [40]

*RW also helped eradicate the background noise that my mind does not yet know how to filter out* [51]*.*

Interesting to note that this sub-category is more present in students’ narratives. While professionals referred to self-reflection practices (probably already acquired in other contexts), students often reported how RW helped them discover reflexivity.

#### Accomplishing learning potential

Our analysis showed how users RW used the technique to “Accomplish learning potential.”

According to the studies’ participants, RWcan enable a learning performancethat would be difficult to reach otherwise. In this context, participants addressed RW as a tool for“accomplishing learning potential.”Within this category, three sub-categories were highlighted: the improvement of skills, personal and professional growth, and assisting the change and development process.

##### Improvement of skills

Participants agreed that the development of skills and abilities through RWwas aimed at their clinical skills and –in relevant areas such as question asking – encouraged reflection and research [35, 46]. Communication skills were also enhanced, as were their relationship with patients, family,colleagues, and friends [35, 38, 46].

Participants said:

“*Through reflective journal writing, my attitude towards learning has changed. I have been encouraged to be a proactive learner. (...) I have been able to identify necessary places for improvement and through research, question asking, goal-setting (...). I have improved my skills in relevant areas”* (student) [35].

*“I feel that it [participation in the study] has been a positive experience by motivating me to improve on my clinical, communication skills, and also my relationships with colleagues, patients, family, and friends*” (professional) [46]

Participants also reported that,in their experience, RWprovided an opportunity to assess and improve themselves and to enhance their self-confidence [38, 40]. Cognitive skills, includinggaining more profoundknowledge and problem-solving, along withtime-management [35, 40, 46, 49], were also enhanced: RW,therefore,represented a learning mode [45].

“*Without reflection, I absolutely believe these skills would be more unattainable for me*”(student) [35]

This sub-category applies more to students’ narratives. Health students mentioned the tools helping them most to develop their skills. Professionals focused principally on what RWcould improve (communication skills or organizational skills).

##### Personal and professional growth

Participantsidentifiedthat RWhad promoted personal [51] and professional growth [35, 46]. RW meant for participants:an ameliorated attitude towards work [46]; a development path for one’s job potential [38]; an enhancement of their introspective knowledge [51]; an enrichment of their expressive capability [38];an improvement of their interpersonal relationships with patients and colleagues [50] and developed their use of critical and reflective thinking [38].

“*Reflecting introduces a new aspect to clinic that focuses on the individual’s learning experience*” (student) [35].

*“I think that it does change the way that you think about the practice of medicine and your own personal tendencies and your interactions with your patients and colleagues. And I think it can be a really powerful driver of culture change*” (professional) [50]

This sub-category is more represented among students than professionals. Students are ‘surprised’ at how important RW was to their learning. Professionals still recognized how RW was an essential driver of change for their clinic activities.

##### Assisting the change and development process

We labeledthe third sub-category“assisting the change and development process.”The changeinvolvedintroducing modifications tothe way of working [48], assessing what needed to be changed to achieve a work-life balance [51], understanding elements that did not allow change, and how to act on them in the future, and also considering new and important issues [46], further information [51] and new ways of thinking. This sub-category equally explained the meaning given to RW by students and professionals.

“*I think writing answer to some of these questions has allowed me to reflect back on the year and think about specific important topics that I might not have thought about again.”*(professional) [46].

(Reflective journaling encouraged) *“Assessing and focusing on the changes that need to be done to achieve the balance in my life and being able to integrate that with my family and in my work as a nurse.”* (Student 16/RJ2) [51]

However, thischange process could not be possible without witnessing change and becoming aware of it [38, 46]. This allowedparticipants to ‘see one’slearning history and path of growth,‘have a picture of the problem, handle things differently, and broadening their vision of the problem [48].

#### Building a philosophical and empathic approach

The “Reflection and reflexivity” category is closely aligned with the “Building a philosophical and empathic approach” category. Participants defined RW as a means for nurturing an intimate and profound level of learning, i.e., a philosophical and empathic approach towards real-life professional issues. The third category consists of three sub-categories: the ability to find benefits in negativity/adversity, assuming an empathetic attitude, and the awareness of things, experiences,emotions.

##### Finding benefits in negativity/adversity

According to participants, RWexerted a therapeutic effect by encouraging professionals and students to focus on the present (43)strictly. It seemed that RWeventually reduced their emotional stress [44, 51]. Likewise,in the contextofnegative experiences [49], its practice acted as a catharsis [46] that could even allow them tolook back at those experiencesafresh – enabling a change in perspective [39].

*“While writing the journal entry, I felt like I was unloading something from inside myself and being set free. This process made me feel better*” (student) [44].

*“It is always good to pause to reflect on my experiences. The most cathartic question was a few months back when I got to describe my really bad experience.”* (professional) [46]

*“Very therapeutic. I wrote on a bad experience, but at the end, we were laughing at it.”* (professional) [49]

This specific approach allowed the practitioner/trainee to improve their self-care and focus on work objectives [51]:

*“Self-reflection and reflective journaling promote self-understanding and is another part of self-care.”* (Student 5/RJ3) [51]

Even if more emerging from students’ voices, professionals appeared genuinely amazed at how learning can be generated out of negativity.

##### Assuming an empathetic attitude

Study participants stressed the fact that RWhelped them develop empathetic attitudes. It seems that RWemphasized the importance of sensitivity and empathy by trying ‘to be in someone else’sshoes,’ especially that of patients or colleagues [36, 37, 44].

*“How reflecting on patient encounters through field notes allowed her to “take a walk in someone else’s shoes*” (student) [36].

*“It helps you see the humanity...*” (professional) [50]

This approach also applied in contexts outside of work and helped the practitioner take off his/her‘white coat’ and understand that before being a professional,he/shewas a person and a human being [36, 37, 46, 50].

“*Which has made me more open to other’s ideas and thoughts*” (professional) [46]

As previously mentioned, according to the participants’ statements, awareness was the cornerstone to effective personal and professional growth [40, 51].

This sub-category is equivalently present among the participants’ groups. Nonetheless, different meaningscould also be highlighted. Students appreciated RWby stressing its value of allowing them to enter deeply ‘into the other’ inner world (mainly patients). Professionals claimed they could recognize the profession’s human and relational aspects, whichcould also be helpful for their extra-professional relationships (family members, friends).

##### Awareness of things, experiences, emotions

Impartially balanced among professionals and students, awareness was cited in terms of ‘how things have affected me rather than simply continuing to work in a robotic manner’ [46], the awareness of who one was and who one has become thanks to the process of change [51]. This professional and relational awareness made it possible to think clearly about one’s practice and the health resources present in the context of belonging [50].

*“Just being aware of what I know now and what I’ll know by the end of the semester … is a great way to learn who I am and what I can change about me for the better.”* (Student 9/RJ1) [51]

The process of awareness that was facilitated by how their RW allowedthem to transform shapeless and straightforward ideasinto words and givethem a specific value and emotional charge [36, 47, 51]: it wasan authentic opportunity to turn emotions and feelings into something tangible –a journey of discovery and personal acceptance [43].

“*After two years or so, when you look back, it’s like, oh,that’s how I was feeling at the time, and right now, I feel differently. There is also this level of satisfaction. Like you have matured out of this thinking*” (professional) [47]

#### Identifying RW feasibility

The fourth category consists of three sub-categories: perceived barriers/impeding factors, facilitating factors, and when and how to use RW. Students and healthcare professionals who had the experience of practicing the RW in their work identified both limitations and facilitating factors and indications about when and how to use RW.

##### Perceived barriers/impeding factors

Some study participants (almost entirely students) identified several barriers to their activity. Some students could not see the benefits and thought RW was a waste of time [35, 38, 51]. However, others, who did see the potential benefits still felt that they lacked the time needed to devote to RW [42] or, sufficient mental space to report and describe a work situation, an excessive similarity of this activity to the regular working practice and, consequently, a lack ofmotivation to write [47, 51]. In addition, some described the strainthey felt in writing down personal/professional experiences [47]. A lack of privacy was another problem, both for the concern about sharing the reflection and for the respect of confidentialityin writing itself [51]. Taken together,it appeared that some study participants did not recognizeRW as an effective means of help [39, 50]. Althoughrealizing the potential of RW,others felt that their tutors did not provide noticeably clearexplanations of the aim of RW– which they would have found useful and motivating [45].

“*To be honest, not a great deal ( … ) it wasn’t really some revelation*” (professional) [50].

“*I got a hard time referring it [my experience] to citations … I could have sat and cried yesterday when I did my essay … when I actually read it [my essay] I thought, oh I don’t know what it means, myself*” (Female 2 - student) [42]

##### Facilitating factors

This sub-category was exclusively interpreted from students’ narratives. They valued the perspectives to use RWin their practice seeing it as a valuable tool to be applied throughout their career [35, 45],with many students reporting that they would continue with this technique [38]. Studentssaw RW as a valuable means of staying focused on their own goals and needs [40, 51]. They remarked that it helped them reduce stress, gain clarity in one’s life and practice [41], and spiritually connect with themselves [45, 51]. Furthermore, RW enabled studentsto discover more information about their health and well-being, ‘it also helped me tie in ideas and beliefs from different sources and relate it to my own’ [51]. RWhelped maintain awareness and recall the medical being/human being dichotomy [37]. It remindedstudentsof the difference between studying literature and refining manual skills and the ability to learn from experience and mistakes [35].

“*During the interview, I felt an element of being more like a ‘normal person’ having a ‘normal conversation’ with another human being. This was a strange realization because it reminded me of the dichotomy that physicians may experience, being doctor versus human*” (student) [37]

##### When and how to use RW

Health professionals (a few) and many students finally mentioned the time considered most appropriate to use RW, underlining its usefulness primarilywas during hardship rather than daily practice [47].Moreover,RWshould not be forced onto someone in any given moment but instead left to individual choice based on one’s spirit of the moment [40, 46].

“.*.. like if you had a patient die; that would be the only time you might write it down*” (professional) [47]

Otherparticipantsconsidered instructions on RW to be too forceful and notapplicable to their own experience of reflection [40]. ‘Reflection wasn’t just signing on the line.’ It allowed constructive feedback for the trainee or the professional. Constructive feedback could be positive or negative, but it was a powerful tool for thinking and examining things [45].

## Discussion

In this meta-synthesis of qualitative studies, we have interpreted the experiences of health professionals and students who used RWduring their education and training. Given the number of studies included, RW users’ experience was predominately investigated in students. This result, although not surprising, raises the question of whether RW in professional training is being used. RW is not used in professional training as often as it is in the academic training of healthcare students.

As to this review’s aim, we could highlight continuities and differences from study participants’ narratives. Our findings offer a conceptualization of usingRW in health care settings. According to the experience of both students (from different disciplines) and health professionals, RW allows its exponents to discover and practice reflectionas a form of cognitive processing [2] and enablethem to develop a better understanding of their lived situation. We also interpreted that RW allows users to make a ‘reflexive journey’ that involves them practicing meta-cognitive skills to challengetheir attitudes, pre-assumptions, prejudices, and habitual actions [24, 26]. This was particularly true for students: “entering personal reflexivity” appears to be newer for them than for the professionals who are likely to acquire reflexivity during academic training. Students seemed more focused on tools than RW-related results. This consideration makes us affirm that reflective capacity is in progress for them.

Challenging pre-assumptions and entering reflexivityenabledRWusers to realize how RW may develop their learning potential to improve skills and personal/professional growth. Skills to be enhanced are quoted mainly by students. Conversely, professionals could comprehend the final purpose of learning, achievable through RW, in terms of communication or organizational abilities. Professionals interpreted skills from RW as abilities to apply in the clinical activities to find new solutions to problems.

The category “Accomplishing learning potential”confirms what many authors highlight: putting thoughts into words not only permits a deeper understanding of events [6], enhances professionalism [52] but also improves personal [16], communication, and empathy skills [4, 17]. In this context, RW fulfills its mandate by letting human sciences [53] and evidence-based health disciplines affect clinical practice. As noted [54], students and health professionals’RW training allowed integrating scientific knowledge with behavioral and sociological sciences to supporttheir learning [55].

Users understood that RWcould be a powerful means of developing empathy and developing their philosophy of care: this consideration is in line with a recent study from Ng and colleagues [24]. Additionally, some authors [4, 17] stressed these empathetic skills and “humanistic”competencies as essential to care for patients effectively [56]. Professionals were amazed how negativity could generate learning through RW. On the other hand, by recognizingand writing experienced negative situations, students could free themselves from feelings impeding empathy.

By employing RW, users reported factors that could encourage or limit its use. These findings further illustrate that RW is not always a tool that is easy to use without adequate training [57]. Almost exclusively, students reported hindering factors (limited time, difficulty in writing and understanding assignments, privacy issues, feeling bored or forced). As to professionals, few describedRW as a very stressful activity. Although students could identify impeding factors, they also recognized many positive ones. For professionals, RW was not to be used every day but in ‘extreme’ situations, requiring reflection and reflexivity to be applied. In general, enhancing motivation to write reflectively [58] should be the first goal of any training to make the process acceptable and profitable for trainees. If this first stage is not accomplished, it will reduce RW’sapparent professional and personal effectiveness among health professionals and students substantially.

### Strengths, limitations, and research relaunches

This review may enrich our knowledge about providing RW as an educative tool for health students and professionals. However, the findings must be applied,taking into account some limitations. We focused our attention only on recent, primary, peer-reviewed studies within the time and publication limits. Qualitative studies often are available as grey literature: considering it may result in a different interpretation of students’ and professionals’ experience in using RW. Therefore, our conceptualization should be read bearing in mind a publication bias and the need to expand the literature search to other sources. Besides limiting the risk of missing published qualitative studies, we reviewed the reference listsof included studies for additional items. Our meta-synthesis is coherent to the interpretation of the included studies’ findings.

At least two reviewers have conducted each step of this systematic review. We purposely did not exclude studies based on a quality assessment to maintain a robust qualitative study sample size and valuable insights.

During analysis, all possible interpretations were screened by authors, and an agreement was reached. Nonetheless, we did not cover all the possible ways to interpret the voices of students and professionals.

Since RW is not used in professional training as often as it is in the academic training of healthcare students, a research relaunch could be investigatingwhether and to what extent RW is being used in in-service training programs. Moreover, the studies included in this review were conducted within Western countries. Students’ and professionals’ perspectives from Africa and Asia are underrepresented within the qualitative literature about experiences of using RW. Therefore, geographicalgeneralizations from the present meta-synthesis should be avoided, and our paper reveals the necessity for RW research in other cultures and settings. Nonetheless, authors of primary studies have paid little attention to cultural and regionaldiversity. Therefore, we recommend furtherinvestigations exploring the differences between cultural backgrounds and howRW is recognized within training programs in different countries. Finally, additional qualitative and quantitative research is required to deepen our understanding of RW’s clinical and psycho-social outcomes in high complexity health practice contexts.

## Conclusion

Our analysis confirms the crucial role of RW in fostering reasoning skills [59] and awareness in clinical situations. While its utility in helping health students and professionals to nurture reflection [2] has been widely theorized, this meta-synthesis provide empirical evidence to support and illustrate this theoretical viewpoint. Finally, we argue that RWis even more critical given the increasing complexity of modern healthcare, requiringprofessionals to develop advanced skills beyond their clinical ones.

### Practical implications

Two important implications can be highlighted:

(i) students and professionals can recognize the potential of RW in learning advanced professional skills. ImplementingRW in academic training as well as continuing professional education is desirable.
(ii) Despite recognizing the effectiveness of RW in healthcare learning, students and professionals may face difficulties in writing reflectively. Trainers should acknowledge and address this.

## Supplementary Information

**Additional file 1:.** Meta-synthesis framework with participants’ narratives.

## Data Availability

The datasets used and/or analyzed during the current study are available from the corresponding author on reasonable request.

## Abbreviations

CASP: Critical appraisal skills programme
ENTREQ: Enhancing transparency in reporting the synthesis of qualitative research
PICo: Population, phenomena of interest and context
PRISMA: Preferred reporting items for systematic reviews and meta-analyses
RW: Reflective writing
