# Supplementary material for: Health professionals and students’ experiences of reflective writing in learning: A qualitative meta-synthesis
Source: BMC Med Educ. 2021 Jul 22;21:394. doi: 10.1186/s12909-021-02831-4 (PMC8299581; doi:10.1186/s12909-021-02831-4)
Supplement: Supplementary file 1 — Additional file 1:. Meta-synthesis framework with participants’ narratives. [file 12909_2021_2831_MOESM1_ESM.docx]

**Meta-synthesis framework with participants’ narratives**

| **Category** | **Sub-category** | **Quotations** |
| --- | --- | --- |
| **REFLECTION AND REFLEXIVITY**  **(Frequency: 10/17)** | **Discovering reflection**  **(Frequency: 5/17)** | “It certainly has forced me to think more about how I have felt during internship” (professional) (35)  “It has given me the chance to reflect on my year periodically. It is easy to become lost in the hustle and bustle, the long hours and the calls during intern year” (professional) (35)  “Being part of this study has made me stop and reflect” (professional) (35)  “This provided opportunity for me to reflect” (professional) (35)  “...writing initiated me to think about my experiences” (professional) (35)  “It has given me the chance to reflect” (professional) (35)  “I think it’s good for physicians to reflect on what we’re doing” (professional) (36)  “When I am presenting my own clinical case, I question myself more often” (professional) (39)  “I ask myself more questions and make more comments to myself ... as if I were my own coach. During an interview, during team meetings, when writing reports, when reflecting back on a past intervention that was successful or difficult” (professional) (39)  “Also made me think during an event because I knew I’d be writing about it later” (student) (38)  “Helped (me) reflect on positive aspects” (student) (38)  “The biggest advantage of the field notes is the fact that some of them made you reflect on topics that you would never have otherwise thought about—or, I should say, really thought thoroughly and reflectively about” (student) (46)  “I don't usually think too much about what happens to me, but through critical reflective journaling, I was able to think carefully about things happening around me. This activity helped me to look into my mind” (student) (37) |
|  | **Entering personal reflexivity**  **(Frequency: 5/17)** | “The questions in this study do make me stop and think about things-how I feel about what I’m doing in residency” (professional) (35)  “This study allowed me to… be an observer of myself this last year” (professional) (35)  “I certainly have become more reflective while writing my responses and I think more cognizant of what those close to me were going through” (professional) (35)  “It’s a nice way to reflect on your clinical practice […] Looking introspectively, see how you can better yourself” (professional) (36)  “Reflective practice is not limited to our professional practice, rather, it becomes self-reflection in our daily activities as an ongoing lived experience” (student) (43)  “Allowing one to attend to specific questions, topics and issues; forcing one to put these things down in a tangible form; permitting one to see when mindful reflective abilities are in their infancy. Journaling helps to eradicate the background noise that my mind does not yet know how to filter out” (student) (43)  “To write the journal, I had to identify and remind myself of what I had done wrong, which made me feel bad. (But) it provided me with an opportunity to reflect on my attitude during the rotation” (student) (37)  “Due to the heavy course load, I rarely look back on my own behaviors during my clinical rotations. But critical reflective journaling helped me to think about the values required to become a caring nurse” (student) (37)  “Helped me ID (identify) my strengths and weaknesses” (student) (38)  “As I was writing a journal, I thought back over my experiences of interacting with patients that day. I also did that with other people. Sometimes something I said or did made patients uncomfortable. Similarly, my fellow students perhaps didn’t like what I said or did, so I had to pay more attention to what I said and did” (student) (41)  “An advantage of journaling is that it helps me look back on my experiences and feelings, re-examine past events, and think back on my image of my own interactions with patients” (student) (41)  “Reflective clinical journaling helped me to revisit time spent in the clinic... so you learn from the experiences rather than just getting through sessions” (student) (40)  “There’s a reason to learn to think and reflect always, as a midwife – that you think about what you might have done differently […]. But for me it’s been an objective with these reflections to see my own personal development, I can probably see two different perspective” (student) (42) |
| **ACCOMPLISHING LEARNING POTENTIAL**  **(Frequency: 13/17)** | **Improvement of skills**  **(Frequency: 9/17)** | “I now remember more about what I did in clinics, which alerts myself about what I need to do further reading, highlights strengths and weaknesses in my clinical performance. I remember my patients better at recall appointments. Reflection encourages me to look up concepts and do further study.” (student) (40)  “Through reflective journal writing, my attitude towards learning has changed. I have been encouraged to be a pro-active learner. Rather than waiting for a tutor to tell me I need to work on certain aspects. I have been able to identify necessary places for improvement and through research, question asking, goal setting, and further reflection, I have improved my skills in relevant areas” (student) (40)  “… and continually strive for improvement. Without reflection, I absolutely believe these skills would be more unattainable for me.” (student) (40)  “(Journal writing) allows me to assess myself and therefore better myself in what I am doing” (student) (38)  “When I read what I had written in the blog and found that my actions had earned the approval of my teachers and patient family members, it gave a big boost to my self-confidence!” (student) (41)  “I could have completed my clinical rotation without putting much thought into it. But, to complete the journal entries, I tried to have more interactions with my assigned patient and to make careful observations” (student) (37)  “Helped to make me feel like I was on track and could manage my time easier, as I did each guideline, I could tick off each task.” (student) (38)  “The CCJ let me understand some of ‘I wanted to let the teacher know that I had done these things, because the teacher probably wasn’t clear on how I had interacted with the patients. The CCJ let me understand some of the problems I had interacting with patients” (student) (41)  “Acknowledged field notes as a way to “practice recording a patient’s story after the fact. I started to notice what things I remember most, what things I tend to write down while interviewing, and how important it is to record these data as soon after talking to the patient as possible. [T]his knowledge helped when I returned to the clinic” (student) (46)  “It led me to resolve the situation in a different way” (student) (45)  “For me, writing is a way of learning. When you talk it’s easy for it to just disappear. When you write you have to think and go through the events and I think that I learn more” (student) (42)  “I feel that it [participation in the study] has been a positive experience by motivating me to improve on my clinical, communication skills, and also my relationships with colleagues, patients, family and friends” (professional) (35)  “In fact, a lot of the negative and positive ways I've felt this last year are easier to remember since I've had to recall them over the months. I've become a fan/rooter for myself by the end... (like it's a sporting event)!” (professional) (35)  “The writing helped me organize and find a solution in building an action plan.” (professional) (44) |
|  | **Personal and professional growth**  **(Frequency: 6/17)** | “… allows them to grow from it in a positive manner and develop with confidence clinically and professionally” (student) (40)  “I was surprised at my own change and growth from the first step to the last. I discovered that compared to the other students my age I became more mature in my thoughts, verbal expression and capabilities” (student) (41)  “It (journaling) can promote personal development” (student) (43)  “The narrative was accurate and had an effect on me. I feel more hopeful, especially after the last few days, which have been particularly bad for me. I have read it two or three times now and I will read it over again probably. It makes me feel less alone” (student) (47)  “Keeping a journal enables me to draw upon my own strength to help others, even those I don’t know” (student) (41)  “Flipping through the journal, I saw that I had received the appreciation of patients and good critiques from instructors. It gave me more motivation to continue to progress!” (student) (41)  “After this semester, I am confident about my ability to write CCJ entries and I will become a reflective caring person. Not only can I care for patients, I can care for others around me” (student) (41)  “Journaling is one tool to reflect, to meditate ourselves. It can promote creativity, self-awareness, and personal development” (student) (43)  “Reflecting introduces a new aspect to clinic that focuses on the individual’s learning experience”  (student) (40)  “I think that it does change the way that you think about the practice of medicine and your own personal tendencies and your interactions with your patients and colleagues. And I think it can be a really powerful driver of culture change” (professional) (36)  “… and therefore, correct my approach and develop a healthy attitude” (professional) (35)  “… and focus on improving myself as an intern” (professional) (35) |
|  | **Assisting the change and development process**  **(Frequency: 6/17)** | Reflective journaling encouraged “assessing and focusing on the changes that need to be done to achieve the balance in my life and being able to integrate that with my family and in my work as a nurse” (student) (43)  “If I write down my bad habits, I am admitting to them, owning them and so I might feel more powerful to change them. I think reflective journaling can put a lot of thoughts in perspective… I also believe that moments of realization can occur through journaling. I might realize why I follow a certain pattern of behavior and then be able to change it” (student) (43)  “Holds me accountable for my changes. Even if I didn’t make the changes that I had hoped to make, it allows me to reflect on why I was unable to change and what I could do in the future differently. Journaling is a valuable tool before it forces the writer to reflect on the decisions they make and explore what changes they can make in the future, while addressing obstacles they may face meeting these goals” (student) (43)  “Good way to look at your professional practice to see how you have improved” (student) (38)  “Up till now, I saw myself mainly as a student. Reflective learning (discussions and journals) helped me to see myself as more of a professional and a practitioner” (student) (40)  “The caring environment for patients in both detailed steps and in the overall process enabled me to see my own learning history and path of growth” (student) (41)  “(and to be) able to see progress” (student) (38)  “Allowed me to take a step back and thoughtfully think about the impact this new information has had on my personal and professional life that I would have otherwise not had an opportunity to consider” (student) (43)  “It forced me to think about my actions, my views, my overall impressions of my experience in a way I may not have otherwise done” (professional) (35)  “This study has helped me to appreciate the various types of ‘progress’ I’ve made during the last year” (professional) (35)  “Participation in this study made me more cognizant of my progress as a clinician throughout this year” (professional) (35)  “I think writing answer to some of these questions has allowed me to reflect back on the year and think about specific important topics that I might not have thought about again” (professional) (35)  “With each email I filled out, I was able to reflect on my recent experiences and assess how I handle things differently now, compared to 3 months ago” (professional) (35)  “You know, it's as if what I find most important now in a case is not what I used to find most important. There are factors we didn't see before. Now, not only do I pay attention to them, but I keep a record of them” (professional) (39)  “I feel that what I am doing is supported by the literature. All my reflections have helped me change my way of working with clients, my approach, my analysis of the work situation and the strategies I use to promote the return to work” (professional) (39)  “It's not the same as listening to the cases of others. I feel it helps in broadening my vision of the problem” (professional) (39) |
| **BUILDING A PHILOSOPHICAL AND EMPATHIC APPROACH**  **(Frequency: 11/17)** | **Finding benefits in negativity/adversity**  **(Frequency: 5/17)** | “It is always good to pause to reflect on my experiences. The most cathartic question was a few months back when I got to describe my really bad experience” (professional) (35)  “Just being able to write what I’m thinking is therapeutic. I spend all day asking people to tell me how they feel... it’s nice to have someone ask me once in a while” (professional) (35)  “Very therapeutic. I wrote on a bad experience but at the end we were laughing at it” (professional) (44)  “Journaling helps with being in the now. Focusing on what you are writing, regardless of the content, can help pull your mind from stress and give energy to what you are thinking at that moment” (student) (43)  “I don’t want to burn out as a nurse and journaling is helping me to see [what] I need to implement for better self-care so I don’t burn out and can be the nurse I want to be” (student) (43)  “They said journaling made them ‘feel like they were released’” (student) (37)  “While writing the journal entry, I felt like I was unloading something from inside myself and being set free. This process made me feel better” (student) (37)  “(Journaling) is beneficial… organizing my thoughts to keep focus on my overall goals, which so far has worked well… I have stayed focused on my self-care and health- promotion goals and even incorporated new holistic practices as the courses progresses” (student) (43)  “Sometimes I vented my anger by writing on the sheet of paper instead of venting it on somebody else and everything stopped. It helped me to continue my day” (student) (45) |
|  | **Assuming an empathetic attitude**  **(Frequency: 5/17)** | “How reflecting on patient encounters through field notes allowed her to “take a walk in someone else’s shoes” (student) (46)  “Writing what happened during the clinical rotation helped me to put myself into the patient's position and to empathize with him” (student) (37)  “It allowed me to connect with the patient in a different format. I felt more focused on emotions and feelings. I enjoyed understanding the patient’s perspective” (student) (47)  “During the interview, I felt an element of being more like a ‘normal person’ having a ‘normal conversation’ with another human being” (student) (47)  “I really just wanted to put onto paper what that patient had been through and what it was like for me to peer into her world and try to understand what she was going through. It was an extremely reflective experience… [and it] also taught me insight into the humanity of doctoring” (student) (46)  “In turn, I might be more apt to stop and think about how/what I’m doing in residency makes my friends/family/fiancé feel” (professional) (35)  “Which has made me more open to other’s ideas and thoughts” (professional) (35)  “It helps you see the humanity...” (professional) (36) |
|  | **Awareness of things, experiences, of emotions**  **(Frequency: 7/17)** | “…how things have affected me rather than simply continuing to work in a robotic manner” (professional) (35)  “After 2 years or so, when you look back, it’s like, oh that’s how I was feeling at the time, and right now I feel differently. There is also this level of satisfaction. Like you have matured out of this thinking” (professional) (48)  “The questions in this study were gentle reminders to me to keep things in perspective. By answering the questions, I was able to remind myself of my priorities, reasons for doing what I’m doing, and primary beliefs and values that I stand for” (professional) (35)  “It is very easy to lose insight into the real reason why we went into this in the first place.” (professional) (35)  “It was like a journey of self-discovery, acceptance, and alignment. […] It was about understanding who I was as an occupational therapist” (professional) (49)  “It’s made me a little more aware of overuse of healthcare resources in general and kind of keep an eye out for it more than I did before…” (professional) (36)  “Just being aware of what I know now and what I’ll know by the end of the semester… is a great way to learn who I am and what I can change about me for the better” (student) (43)  “[F]ield notes can be an effective tool for deeper and more purposeful reflection. Writing thoughts and reflections require[s] students to put words to what are often unformed ideas or thoughts and require[s] more concentration than [does] simply vocalizing.” (student) (46)  “[W]eek to week, I realized how much more open I started to become about writing about my experiences… I would say that in the past I’ve kept many of my feelings and painful experiences to myself. It’s been very rare, until this year, for me to be so reflective and in touch with my emotions and thoughts” (student) (46)  “Self-reflection assists us to understand and know ourselves more fully on our own personal beliefs and values. More positive feelings such as hope, love and forgiveness may also be revealed during this stage of reflection…” (student) (43)  “Self-reflection and reflective journaling promote self-understanding and is another part of self-care” (student) (43)  “Field notes,” another student stated, “are an opportunity to take all those mixed emotions (like how you felt the first time a patient broke down and cried while talking to you) and put [them] down on paper, where it makes those feelings real” (student) (46)  “This is the area that field notes were most beneficial for me, in thinking about what kind of doctor I want to be and how I want to interact with patients” (student) (46)  “Because it allows you to think clearly about your practice” (student) (38) |
| **identifying RW feasibility**  **(Frequency: 12/17)** | **Perceived barriers/impeding factors**  **(Frequency: 10/17)** | “[Reflective writing] takes up quite a lot of valuable time that could be used for marked assessments in Semester 2” (student) (40)  “The only negative aspect of reflective learning is that it can be a little time consuming…” (student) (40)  “The instructor modeled how to care for the patient right at the bedside. She chatted with the patient and told me to stand at the side and listen. Afterwards she asked me to go back and write about the process, skills, and reflections (experience) of chatting with the patient at the bedside. This was the most difficult thing I had to learn in my Psych practicum. The teacher helped me overcome that bottleneck” (student) (41)  “I didn’t understand the journal caring topics and table of definitions and didn’t know what to write initially” (student) (41)  “Usually, the journal was submitted in the second and third week of that month. That was the time that most of our reports needed to be done. When you were feeling you didn’t have time to finish all your reports, you still had to write this thing, but you were so tired, you’d be so angry” (student) (41)  “In my opinion the problem is that the journal provides no tangible help for students. It’s a piece of paper: I help myself. What kind of help is it?” (student) (45)  “I think the problem is a lot of us don’t get time [off] from our work as well” (student) (50)  “And also, we do nights, the nights are nightmares, you can’t do anything in daytime ‘cos you won’t be able to concentrate” (student) (50)  “There’s a lot to cover... there’s a lot of work, and a lot of different sections… I think that I procrastinated, how much work I need to put into this, as in, how much research I needed to do...and the number of hours needed to put into it… I massively underestimated it” (student) (50)  “Yeah, ‘cos you’re reading loads, you’re not just looking at one thing, you’re looking at loads of things that have got to go into it” (student) (50)  “…because it’s a reflective study you’re having to leave a lot till late on aren’t you [murmured agreement] you know there’s not much time…” (student) (50)  “I purposely haven’t booked my appointment with my academic supervisor yet, ‘cos I don’t feel I am at a stage yet where I can gain enough benefit from their input, plus the fact that they are only allowed to look at 500 words” (student) (50)  “I got a hard time referring it [my experience] to citations… I could have sat and cried yesterday when I did my essay… when I actually read it [my essay] I thought, oh I don’t know what it means, myself” (student) (50)  “I really do not believe that students, faced with the requirement to produce reflective writing for assessment within the current education system, are free to produce a ‘real’ or ‘true narrative’...” (student) (50)  “It is waste of time” (student) (43)  “Boring” (student) (43)  “Fearful to disclose thoughts and feelings” (student) (43)  “Invasion of privacy” (student) (43)  “I am an introvert and prefer to keep my feelings and reflections personal. I am a new nurse. I graduated a year ago and have been working for roughly eight months. I have spent that time trying so hard to prove I am a nurse and can do this job that reflecting on things I need to do to improve in my practice and reflecting on things I could have done better is daunting” (student) (43)  “Even when I didn't have any particular instance to reflect on, I had to find an event because journaling was part of required assignments. This made me wonder what the journaling was for. I felt I was writing it just to meet the course requirement” (student) (37)  “The structured format prevented me from writing freely about my thoughts. I would have felt more comfortable if there was no requirement in terms of its format, like a personal diary” (student) (37)  “I've had a hard time figuring out what I was supposed to do in the situation (that I reflected upon), and I am still not sure about it. I wish I had a chance to share my experiences with others and have their opinions” (student) (37)  “Not sure what they wanted us to write” (student) (38)  “Too time consuming” (student) (38)  “No point, I learn from experiences” (student) (38)  “I think I can reflect personally without using a journal” (student) (38)  “Too much effort” (student) (38)  “I thought it was good to write, but maybe a clearer aim, a bit more for the supervisors because though some understood the background to the idea and felt that it was important, many thought – signing on the line, that’s not so much – we’ve talked about this” (student) (42)  “To be honest, not a great deal […] it wasn’t really some revelation” (professional) (36)  “I think it’s too much like work. I couldn’t be bothered” (professional) (48)  “I think if it’s very stressful, like if you had a patient die” (professional) (48) |
|  | **Facilitating factors**  **(Frequency: 8/17)** | “However, considering the benefits, it is definitely a valuable tool for our long-term career” (student) (40)  “The best result of writing a journal was the encouragement of the instructor. In the clinic, we were frequently criticized, so perhaps our only encouragement came at that time. That gave me motivation to continue caring for patients and to continue writing a journal!” (student) (41)  “When I read what I had written in the blog and found that my actions had earned the approval of my teachers and patient family members” (student) (41)  “Although I learn best from structured and flowing, comprehensive logic mostly found in books, many things are not learnt through reading. Some things that occur can be labelled as ‘experience’ or ‘wisdom’ that sometimes only going through the difficulty will give rise to the understanding and empathy for the other party… However, the experience and understanding of interlinked complexities of human learning and habits to the dental environment was beneficial in a manner of becoming aware of such variations from textbook learning” (student) (51)  “One participant reported that it was helpful ‘to have some guidance in the form of questions also, because I became more focused on the goal at hand’” (student) (43)  “I find doing reflective learning in a discipline that is so focused on scientific and evidence-based reasoning a refreshing experience” (student) (40)  “I spoke about the team meeting: it was something new, different from routine.” (student) (45)  “Journaling is a new strategy for my self-care that look forward to. Journaling allows me to reduce stress, focus on my needs, gain clarity in my life, and connect spiritually with myself” (student) (43)  “Because it allows you to think clearly about your practice” (student) (38)  “Reflective journaling has helped me gain more insight into my health and well-being. It also helped me tie in ideas and beliefs from different sources and relate it to my own” (student) (43)  “(During the interview, I felt an element of being more like a ‘normal person’ having a ‘normal conversation’ with another human being). This was a strange realization because it reminded me of the dichotomy that physicians may experience, being doctor versus human” (student) (47)  “Reflective learning should absolutely be included in future courses. There is a vast difference between being able to study the literature and hone manual skills and being able to learn from experiences, mistakes, ask for help, self-evaluate” (student) (40)  “It’s good to write honestly […]. I’ve had that kind of comment when I’ve felt useless […]” (student) (42)  “It was a bit like this for me too, at the end… a bit hounded… I have to write, have to write – those damned reflections… and then when it’s done so, phew! It’s quite nice to have it done” (student) (42)  “I didn’t really understand that it was supposed to be an instrument for my own sake. I didn’t really understand that at the beginning” (student) (42) |
|  | **When and how to use RW**  **(Frequency: 4/17)** | “…like if you had a patient die; that would be the only time you might write it down” (professional) (48)  “More key incidents. Not every day.” (professional) (48)  “Keeping a journal should be recommended and not required” (student) (45)  “Very general guidelines therefore able to freely write entries” (student) (38)  “I don’t need instructions on reflecting my own experiences” (student) (38)  “What I thought was good at the end was – what happened? What could I have done differently? I didn’t always write this, but I thought about it and I’ll carry on thinking like this… this is what happened…” (student) (42)  “Reflection wasn’t just signing on the line – it was a matter of going through things […] things to think about even after I’d been given feedback” (student) (42)  “I think that no matter how you are, or how you do or how well you perform you should be given feedback – giving constructive feedback means giving both positive and negative feedback…” (student) (42) |
